# A hidden cost of migration? Innate immune function versus antioxidant defense

**DOI:** 10.1002/ece3.3756

**Published:** 2018-02-07

**Authors:** Cas Eikenaar, Caroline Isaksson, Arne Hegemann

**Affiliations:** ^1^ Institute of Avian Research Wilhelmshaven Germany; ^2^ Department of Biology Lund University Lund Sweden

**Keywords:** avian migration, ecophysiology, immunity, oxidative stress, trade‐off

## Abstract

Migration is energetically demanding and physiologically challenging. Migrating birds, for example, need to boost their antioxidant defenses to defeat the pro‐oxidants produced during high energetic activity. The enhanced antioxidant defense possibly withdraws limited resources (e.g., energy or micronutrients) from other physiological functions, such as immune defense. Such a trade‐off might not occur outside the migration seasons or in resident individuals. Here, we investigate whether there is a negative relationship between innate immune function and antioxidant defense by sampling both migrating and resident blackbirds (*Turdus merula*) at the same location during the same period of the annual cycle. We show that in migrating blackbirds microbial killing capacity (BKA), an integrative measure of baseline innate immune function was negatively correlated with total nonenzymatic antioxidant capacity. In contrast, in resident conspecifics, sampled at the same time and location, these two physiological measures were not correlated. This suggests that migrating birds trade off innate immune function and antioxidant defense. Furthermore, and likely a consequence of this trade‐off, in migrant blackbirds BKA was positively correlated with oxidative damage to lipids. In resident blackbirds BKA and degree of lipid oxidation were uncorrelated. The mechanism and currencies of the supposed trade‐off are currently unknown, but energetic investments or micronutrients are likely candidates. Future experimental studies could provide more conclusive evidence for this trade‐off; yet, our results open up a new level of thinking about the physiological costs of migration.

## 1 INTRODUCTION

Central to life‐history theory is the idea that organisms are resource limited; hence, investment of resources in one trait reduces the possibility to invest resources in other traits (Roff, 1992; Stearns, 1992). While resulting physiological trade‐offs could be masked by between‐individual variation in resource acquisition (e.g., van Noordwijk & de Jong, 1986), they are most likely to be apparent during energetically or physiologically demanding periods (Schwenke, Lazzaro, & Wolfner, 2016; Wone, Ojha, Contreras, & Davidowitz, 2014). Migrating animals face high energetic demands (Bairlein et al., 2015; Schmaljohann, Fox, & Bairlein, 2012; Wikelski et al., 2003), which appears to elevate the levels of pro‐oxidants (Costantini, Cardinale, & Carere, 2007; Jenni‐Eiermann, Jenni, Smith, & Costantini, 2014), likely via leakage of reactive oxygen species (ROS) from the mitochondrial membrane as a consequence of a higher metabolic rate. The ROS are highly reactive and cause oxidative damage to biomolecules such as proteins and lipids, which in the long run affects cellular function and ultimately, fitness (Ouyang, Lendvai, Moore, Bonier, & Haussmann, 2016). Animals can counteract pro‐oxidants by boosting their antioxidant defenses (both the production as well as the consumption of antioxidants); however, this may not be cost‐free (Monaghan, Metcalfe, & Torres, 2009). An upregulated antioxidant defense could take away limited resources (e.g., energy or micronutrients) from other functions such as reproduction, lipid storage, or immune defense (Evans & Halliwell, 2001; Griboff et al., 2014; Isaksson, Sheldon, & Uller, 2011). Regarding immune defense, it has been hypothesized that migrating animals should boost their immune function during migratory periods in order to be prepared for more and/or novel pathogens that they may encounter during their travels (Buehler, Tieleman, & Piersma, 2010; Møller & Erritzoe, 1998). Recently though a study on a partial migrant, the common blackbird (*Turdus merula*, blackbird hereafter), showed that migrating individuals had lower microbial killing capacity than sedentary conspecifics sampled at the same location during the same period (Eikenaar & Hegemann, 2016). Microbial killing capacity is an integrative measure of baseline innate immune function (French & Neuman‐Lee, 2012; Millet, Bennett, Lee, Hau, & Klasing, 2007; Tieleman, Williams, Ricklefs, & Klasing, 2005), an animal's first line of defense against foreign harmful bacteria, viruses, and other pathogens. The study of Eikenaar and Hegemann (2016) thus supports the hypothesis that birds may be forced to compromise immune function during physiologically demanding migrations (Buehler & Piersma, 2008). Interestingly, in the same blackbird study system, migrating individuals had higher nonenzymatic antioxidant capacities than sedentary individuals, that is migrants produce or ingest more antioxidants, suggesting that they boost their antioxidants to counteract the increased pro‐oxidant exposure (Eikenaar, Källstig, Andersson, Herrera‐Dueñas, & Isaksson, 2017). The antioxidant defenses were then highly efficient as migrating and sedentary individuals did not differ in oxidative damage to lipids (Eikenaar et al., 2017). Together, these data suggest that due to elevated energetic and physiological demands, migrants may trade off investment in baseline innate immune function against investment in antioxidant defense. However, to show such a trade‐off, the relationship between these two physiological traits should be determined within individual birds, and clear hypotheses about their functional interaction should be provided (Zera & Harshman, 2001).

In this study, we do so by relating the microbial killing capacity (hereafter BKA) to total nonenzymatic antioxidant capacity (hereafter AOX) within individual blackbirds. By sampling both migrating and sedentary blackbirds at the same location during the same period of the annual cycle, we were able to determine whether there is a negative correlation between these two physiological traits which would suggest a functional interaction as a consequence of migration. In addition to AOX, we determined the birds' malondialdehyde (MDA) concentration, a commonly used biomarker of oxidative damage to lipids. Our previous work has also shown that haptoglobin‐like activity, another measure of innate immune function, differs between sedentary and migratory birds (Eikenaar & Hegemann, 2016). Haptoglobin, however, also has antioxidant properties in that it can bind hemoglobin, a mechanism that may be important for migrating birds engaging in endurance flight (Nebel et al., 2012). With this bimodal physiological function, we could not formulate a clear hypothesis as to whether or not migrants trade off haptoglobin synthesis with antioxidant defense. Hence, in this study, we focused on BKA as an integrative measure of baseline innate immune function.

We expected that migrating blackbirds trade off investment in BKA with investment in AOX and thus predicted a negative relationship between these two physiological parameters. As a consequence of this trade‐off, we expected BKA and MDA concentrations to be positively correlated. In contrast, we expected that resident blackbirds, which do not face the energetic and physiological challenges of migration, are not forced to trade off investment in BKA against investment in AOX. For residents, we thus predicted that BKA is not related to AOX or to MDA concentration.

## 2 MATERIAL AND METHODS

### 2.1 Field procedures

The study was conducted on Helgoland Island (54°11′N, 07°55′E, 0–60 m elevation), a small (<1 km^2^) isolated island in the German Bay of the North Sea. The birds were caught in October 2014, during the autumn migration period of this species, when hundreds of blackbirds use the island as a stopover site and mix with the local, sedentary blackbirds (Dierschke, Dierschke, Hüppop, Hüppop, & Jachmann, 2011; Sacher, 2009). Most blackbirds caught during stopover on Helgoland winter in the United Kingdom and breed in Scandinavia (Dierschke et al., 2011). Upon capture, the birds were blood‐sampled from the wing vein within 10 min. Samples were placed on ice for <4 hr, after which the plasma was separated by centrifugation and frozen at −20°C until laboratory analyses. After sampling, birds were aged (1st year or adult) and sexed on plumage after Svensson (1992) and fat stores were scored on a scale from 0 (no fat) to 8 (furcular and abdomen bulging, and breast covered with fat) following Kaiser (1993). All birds were trapped during daylight hours (7 a.m.–6 p.m.). All procedures were approved by the Ministry for Agriculture, the Environment, and Rural Areas, Schleswig‐Holstein, Germany.

### 2.2 Assignment of migratory status

All birds received a metal ring and a unique combination of four color rings for later identification in the field. Weather permitting, daily searches for color‐ringed birds were made throughout October and November. Helgoland is tiny (1 km^2^) and birds can easily be resighted if they stay on the island. Migrants were separated from residents combining two approaches (Eikenaar & Hegemann, 2016; Eikenaar, Müller, Klinner, & Bairlein, 2015; Eikenaar et al., 2017). First, we assumed that 10 blackbirds ringed (with a metal ring only) on Helgoland in previous breeding seasons and retrapped by us in autumn were Helgoland residents. This assumption rests on a radio‐telemetry study showing that most (91%) Helgoland blackbirds are sedentary (Sacher, 2009). The assumption seems valid because 9 of the 10 birds from this category were resighted on Helgoland several weeks after we color‐ringed them (the one exception was resighted only once, after 8 days). Second, 10 newly caught birds were considered resident because they were resighted (or retrapped) more than 19 days after our color‐ringing. We chose 19 days as a cut‐off point, instead of 9 days as in Eikenaar and Hegemann (2016) and Eikenaar et al. (2017), because a very recent radio‐telemetry study on Helgoland, revealed that the longest stopover made by migrating blackbirds was 19 days (median stopover duration was 4 days, *n* = 57, F. Müller, unpublished data). The 10 newly ringed residents were usually resighted (or retrapped) rapidly and multiple times after color‐ringing (median number of days until first resighting was 1.5 days (range: 0–13 days), and the median number of their resightings during the study period was 8 observations (range: 2–18 observations)). Moreover, 14 of the 20 birds we assigned as residents were again seen on Helgoland in the spring of 2015, boosting our confidence in the correct assignment as residents. Newly caught birds that were never resighted were considered migrants (*n* = 35). Ten newly caught birds that were resighted only within 19 days of color‐ringing (range: 1–11 days) have most likely been migrants. Yet, to reduce the possibility of misassignment in status and to make our assignment as conservative as possible, we excluded these birds from this study (after Eikenaar et al., 2017). The fact that immigration and emigration rates in the Helgoland blackbird breeding population are very low (Sacher, 2009), increases the likelihood of accurate assignment of status.

### 2.3 Laboratory work

All physiological parameters were determined from plasma samples. AOX was measured using the ferric reducing antioxidant power (FRAP) assay, which gives the overall reducing potential, that is the nonenzymatic antioxidant potential of the sample (Benzie & Strain, 1996). The use of a global measure like the FRAP assay can in some instances be preferred over specific markers. Here, for example, we are interested in the total antioxidant power as a functional trait rather than correlations with specific antioxidants which are likely to reveal different patterns due to compensatory mechanisms among the antioxidants, thus difficult to interpret in relation to immune function (Monaghan et al., 2009). As the FRAP assay is affected by uric acid (UA) concentration (Benzie & Strain, 1996), UA concentration was assessed using a commercial kit from SPINREACT (Sant Esteve de Bas, Spain). One of the major damages that occur as a result of ROS‐induced oxidative stress is lipid peroxidation (Costantini, 2014). The main products of lipid peroxidation are hydroperoxides, which further break down into secondary metabolites such as aldehydes, alcohols, and ketones (Gray, 1978). Malondialdehyde (MDA), a secondary product of peroxidation of polyunsaturated fatty acids (Gardner, 1979), is the most frequently used biomarker of overall lipid peroxidation level. MDA concentration was measured after Eikenaar et al. (2017) by coupled gas chromatography and electron ionization mass spectrometry (GC/EI/MS) analysis after derivatization with O‐(2,3,4,5,6‐pentafluorbenzyl) hydroxylamine hydrochloride (PFBHA·HCl). BKA (against *Escherichia coli*) was determined following the method described by French and Neuman‐Lee (2012) with a few modifications (see Eikenaar & Hegemann, 2016). Specifically, we used a dilution of 3 μl plasma mixed in 4 μl of 10^5^ *E. coli* solution. This concentration is based on validation tests we ran earlier. We measured bacteria growth at 600 nm using a microplate reader (see Eikenaar & Hegemann, 2016). The BKA assay provides an integrative measure of baseline innate immune function and does not require an immune challenge. It involves phagocytosis (e.g., by macrophages, heterophils, and thrombocytes), opsonizing proteins like complement and acute phase proteins, and natural antibodies (which are unaffected by previous exposure; Ochsenbein & Zinkernagel, 2000). Compared to other measures of baseline immune function, one of the primary benefits of the bacteria‐killing assay is that it quantifies the ability of an organism to remove a pathogen that could be encountered in the wild. Furthermore, several immune components are measured simultaneously. Killing capacity of plasma against *E. coli* as applied in this study reflects complement activity but also requires phagocytosis and the presence of natural antibodies. Natural antibodie are unique immunoglobulin molecules, their presence does not require previous exposure to a particular antigen, they are found in naive animals, including those raised in germ‐free environments and are encoded directly by the genome (Matson, Tieleman, & Klasing, 2006; Ochsenbein & Zinkernagel, 2000; and references therein). Hence, the bacterial killing assay provides a rather integrative measure of baseline innate immune function (French & Neuman‐Lee, 2012; Millet et al., 2007; Tieleman et al., 2005). Details of all laboratory work can be found in the Appendix S1. Note that because for some individual birds, the plasma volume was insufficient to run all assays, the sample sizes of this study are lower than those in Eikenaar and Hegemann (2016) and Eikenaar et al. (2017).

### 2.4 Data analysis

FRAP assays are commonly corrected for UA concentration (e.g., Cram, Blount, York, & Young, 2015; Eikenaar et al., 2017; Kilgas et al., 2010; Romero‐Haro & Alonso‐Alvarez, 2014), although UA has antioxidant properties, at least in vitro (Stinefelt, Leonard, Blemings, Shi, & Klandorf, 2005). This correction is necessary because, with UA formed in the bird's body by catabolism of proteins, its plasmatic concentration changes rapidly after feeding and during fasting (e.g., Alonso‐Alvarez & Ferrer, 2001; Cohen, Klasing, & Ricklefs, 2007; Geiger, Kauffmann, Le Maho, Robin, & Criscuolo, 2012; Kolmstetter & Ramsay, 2000), which affects the FRAP measurements (Benzie & Strain, 1996; Cohen et al., 2007). In both migrant and resident blackbirds sampled on Helgoland, UA concentrations are high in the early morning and decrease during the day, probably reflecting either a diurnal decrease in food intake or a shift in diet during the day (Eikenaar et al., 2017). UA has been estimated to contribute 60% to FRAP values (Benzie & Strain, 1996), and in the current dataset, AOX is strongly correlated with UA concentration (see Supporting information). Hence, diurnal variation in UA concentration strongly biases the blackbirds' AOX values (Eikenaar et al., 2017), which could mask a relationship between AOX and BKA. We, therefore, corrected AOX for UA using the unstandardized residuals of the linear regression of UA concentration on AOX (following Kilgas et al., 2010; Cram et al., 2015). This correction effectively eliminates the bias in AOX that otherwise results from diurnal variation in UA concentration (Eikenaar et al., 2017, table 2). AOX was log_10_‐transformed prior to this correction. Although plasmatic lipid concentrations can have confounding effects on measures of lipid peroxidation (Pérez‐Rodríguez et al., 2015), in this study, MDA concentration was not corrected for fatty acid concentrations (see Supporting information for rationale).

To investigate whether UA‐corrected AOX and MDA concentration were related with BKA in migrants, but not residents, we used general linear models with status (migrant or resident), BKA, and their interaction as independent variables. In birds, markers of the oxidative balance may show diurnal variations, vary with fat stores, and differ among age classes and between the sexes (e.g., Alonso‐Álvarez, Pérez‐Rodriguez, García, Viñuela, & Mateo, 2010; Eikenaar et al., 2017; Jenni‐Eiermann et al., 2014; Skrip, Seeram, Yuan, Ma, & McWilliams, 2016; Skrip et al., 2015; Van de Crommenacker, Komdeur, Burke, & Richardson, 2011). To take into account, their potentially confounding or masking effects, time of capture, fat score, age (1st year or adult), and sex were entered as covariates into all models. Model selection was carried out using stepwise backward elimination of nonsignificant terms (*p *> .05) in order of least significance. We also checked for interactions between status and time of capture, fat score, and age, but as none was significant (all *p* > .33), these were again removed from the models. BKA was standardized prior to analyses to facilitate interpretation of its regression coefficient (Schielzeth, 2010). To reduce the number of zeros behind the decimal point in the parameter estimates, BKA was divided by 1,000. Note that in the figures, original values of BKA are presented. To achieve normality of residuals, MDA concentration was log_10_‐transformed prior to analysis.

In the dataset on migrating blackbirds, transformation of BKA values failed to normalize the data. Therefore, we used Spearman rank correlations as posthoc tests to investigate the relationships between physiological parameters within migrants and within residents. For these nonparametric posthoc tests, we used nontransformed data. All analyses were performed in SPSS v. 23.0 (IBM, New York).

## 3 RESULTS

**Figure 1:**
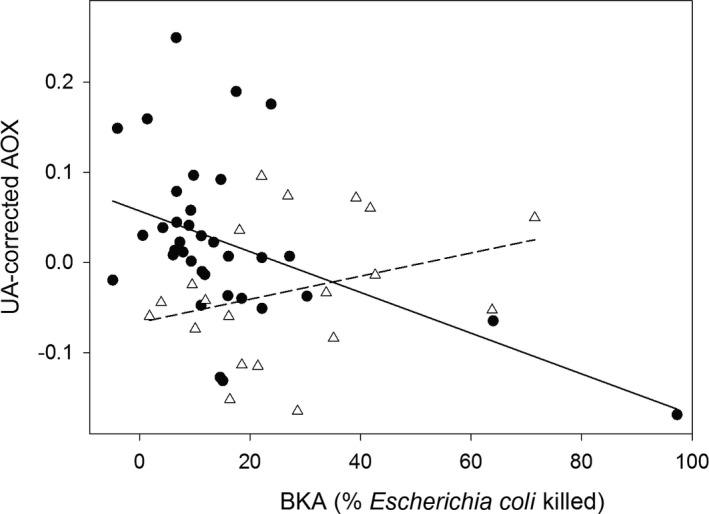
The relationship between microbial killing capacity (BKA) and total nonenzymatic antioxidant capacity corrected for uric acid concentration (UA‐corrected AOX) in migrating (solid circles, *n* = 35) and resident (open triangles, *n* = 20) common blackbirds. The solid line (migrants) and dashed line (residents) serve to illustrate the interaction between status and BKA. Note that when excluding the migrant with the very high BKA, the interaction between status and BKA remains significant (also see Section 2.1)

Migrants had lower BKA than residents (Mann–Whitney *U*‐test: *Z* = −2.92, *p* = .003, *n* = 55). UA‐corrected AOX differed between resident and migrant conspecifics depending on their BKA level as indicated by the significant interaction between BKA and status (Table 1, Figure 1). Posthoc tests showed that in migrating blackbirds, BKA was negatively correlated with UA‐corrected AOX (rho = −0.48, *p* = .004, *n* = 35, Figure 1), whereas in resident blackbirds, these two physiological measures were not correlated (rho = 0.31, *p* = .18, *n* = 20, Figure 1). Fatter individuals tended to have lower UA‐corrected AOX than lean individuals (Table 1). Time of capture, age, and sex did not affect UA‐corrected AOX (Table 1). UA concentration was not correlated with BKA, neither in migrants (rho = 0.07, *p* = .71, *n* = 35) nor in residents (rho = −0.02, *p* = .94, *n* = 20).

**Table 1:** Effects of microbial killing capacity (BKA), status (migrant or resident), their interaction, time of capture, fat score, age, and sex on total nonenzymatic antioxidant capacity corrected for uric acid concentration (UA‐corrected AOX), and lipid peroxidation (malondialdehyde (MDA) concentration) in common blackbirds (*N* = 20 resident and 35 migrants). Variable statistics are given as in the step prior to removal from the model. The final models are in bold. For each variable, the *df* = 1

Variable	UA‐corrected AOX			MDA concentration		
	β ± *SE*	*t*	*p*	β ± *SE*	*t*	*p*
BKA	**1.28 ± 0.97**	**1.32**	**.19**	**−4.65 ± 4.93**	**−0.94**	**.35**
Status	**0.05 ± 0.02**	**2.35**	**.023**	**0.10 ± 0.12**	**0.84**	**.40**
BKA × status	**−3.54 ± 1.21**	**−2.93**	**.005**	**17.48 ± 6.13**	**2.85**	**.006**
Time of capture	0.001 ± 0.001	1.50	.14	**−0.012 ± 0.004**	**−3.21**	**.002**
Fat score	−0.021 ± 0.012	−1.77	.083	0.072 ± 0.062	1.17	.25
Age	0.003 ± 0.024	0.14	.89	−0.23 ± 0.12	−1.94	.058
Sex	0.022 ± 0.022	0.99	.33	−0.018 ± 0.115	−0.16	.87

Reference categories are resident for status 1st year for age and male for sex.

**Figure 2:**
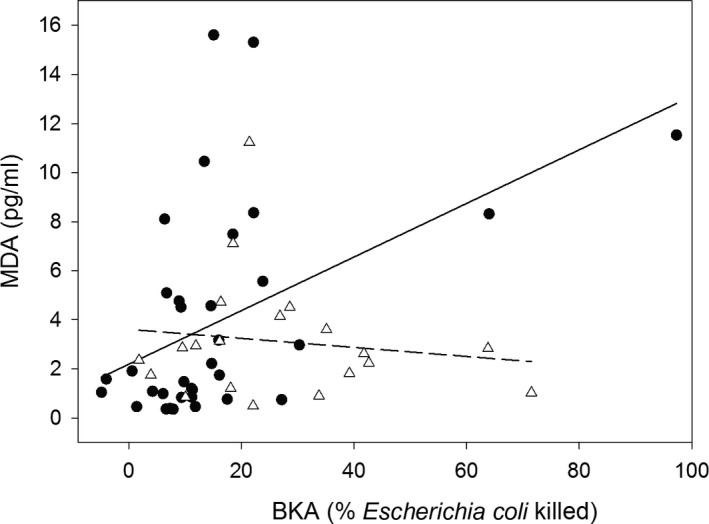
The relationship between microbial killing capacity (BKA) and malondialdehyde (MDA) concentration in migrating (solid circles, *n* = 35) and resident (open triangles, *n* = 20) common blackbirds. The solid line (migrants) and dashed line (residents) serve to illustrate the interaction between status and BKA. Note that when excluding the migrant with the very high BKA, the interaction between status and BKA remains significant (also see Section 2.1)

In the model on MDA concentration, there also was a significant interaction between BKA and status (Table 1, Figure 2); hence, the relationship between BKA and MDA concentration differed between residents and migrants. Posthoc tests showed that in migrating blackbirds, BKA was positively correlated with MDA concentration (rho = 0.48, *p* = .004, *n* = 35, Figure 2), while in resident blackbirds, BKA and MDA concentrations were not correlated (rho = −0.07, *p* = .76, *n* = 20, Figure 2). Time of capture had a negative effect on MDA concentration, that is, MDA concentrations decreased over the day, and 1st year birds tended to have higher MDA concentration than adult birds (Table 1). Fat stores and sex did not affect MDA concentration (Table 1).

One bird, a migrant, had much higher BKA than the other birds in our dataset. Excluding this individual did not qualitatively change our results; in the final models, the interaction between status and BKA remained significant in both the AOX and MDA models (β ± *SE* = −3.43 ± 1.50, *t* = −2.28, *p* = .027 and β ± *SE* = 23.38 ± 7.51, *t* = 3.11, *p* = .003, respectively). Additionally, we have no biological reason to exclude this data point.

## 4 DISCUSSION

We found that in migrating blackbirds microbial killing capacity, an integrative measure of baseline innate immune function was negatively correlated with nonenzymatic antioxidant defense. In the less energetically and physiologically challenged resident blackbirds, no such correlation was detected. This strongly suggests that migrating birds trade off investment in innate immune function and antioxidant defense. Additionally, we found that oxidative damage to lipids increased with microbial killing capacity in migrants, but not in residents. These findings add a new level of understanding to the physiological costs of migration.

Our UA‐corrected measure of antioxidant defense includes both endogenously produced nonenzymatic antioxidants, such as thiols and ascorbic acid (Vitamin C), and exogenous dietary nonenzymatic antioxidants, such as flavonoids, alfa‐tocopherol (Vitamin E), and carotenoids. It thus represents an animal's investment in antioxidant defense, that is, the resources invested to produce antioxidants and/or to search for and consume antioxidant‐rich food. Migrant birds may selectively forage on antioxidant‐rich fruits (Bolser et al., 2013). However, as endogenously synthesized antioxidants contribute more to AOX measured in FRAP assays than do dietary antioxidants (Benzie & Strain, 1996; Chen et al., 2011), in our study, the production of nonenzymatic antioxidants probably was the migrants' main investment in antioxidant defense. The currency (or currencies) of this investment are currently not well defined. A study on the freshwater shrimp *Palaemonetes argentines* found that activation of the antioxidant response decreased lipid storage, indicating that the antioxidant defense is energetically costly (Griboff et al., 2014). Still, whether the energetic investment in antioxidant defense is substantial enough to result in a trade‐off with innate immune function remains to be investigated. Alternatively (or additionally) to an energetic trade‐off occurring between AOX and BKA, the two systems may compete over micronutrients, for example for macrophage function (Erickson, Median, & Hubbard, 2000) or for the synthesis of nonenzymatic antioxidants, such as glutathione.

Irrespective of its currency, a physiological trade‐off between innate immune function and antioxidant defenses would inevitably carry physiological costs that could affect a migrant's fitness. For the immediate defense against foreign harmful viruses, bacteria, and other pathogens, an animal needs a properly maintained and functioning baseline innate immune system. Reducing investment in baseline innate immune function could thus be detrimental for a migrant's health and reduce its survival probabilities (Hegemann, Marra, & Tieleman, 2015; Hegemann, Matson, Versteegh, Villegas, & Tieleman, 2013). On the other hand, investment in immune defense at the expense of nonenzymatic antioxidant defenses likely will increase the oxidative damage a migrant incurs. The positive correlation between BKA and MDA concentration in migrating individuals very likely illustrates this second cost; individuals maintaining high baseline innate immune function at the expense of investment in nonenzymatic antioxidant defenses suffered much oxidative damage to lipids. Our findings may appear to be conflicting, but are not, with findings from a recent paper which suggests that wild birds can mount an immune response without suffering systematic oxidative stress (Cram et al., 2015). First, the study by Cram et al. (2015) was performed during a quiescent period of the annual cycle, and the authors themselves suggest that a trade‐off between immune function and antioxidant defenses might only be visible during a demanding season. Second, while their study used a local immune challenge to trigger an immune response, we investigated baseline immune function. These two are differently regulated and need to be studied separately (Hegemann, Matson, Both, & Tieleman, 2012; Hegemann et al., 2013). A positive association between BKA and MDA concentration could have also resulted from immune cells releasing pro‐oxidants (oxidative burst), a process that is known to occur when birds mount an immune response (e.g., Von Schantz, Bensch, Grahn, Hasselquist, & Wittzell, 1999). If this would also happen as part of maintaining baseline innate immune function, this process could have contributed to the positive relationship we observed between BKA and MDA concentration in migrants. This, however, is unlikely as in resident blackbirds MDA concentration clearly did not increase with BKA (Figure 2b). Oxidative bursts could also cause a negative association between immune function and antioxidant defense, if, to enhance the effect of oxidative bursts, a downregulation of the antioxidant defenses would take place. In other words, a trade‐off between somatic maintenance and direct survival as a consequence of infection may be an alternative explanation for the negative association between AOX and BKA observed in migrants, rather than a trade‐off based on limited resources. We, however, have several reasons why this explanation is unlikely. First, BKA is thought to be a poor indicator of current parasitemia (Matson et al., 2006). Second, residents were caught and sampled in the same very small spatial area (<1 km^2^) as migrants, and consequently, the two groups experienced the same local pathogen pressure and similar needs for oxidative bursts. Still, as we did not measure pathogen or parasite infections directly, there is a possibility that residents and migrants have different infections because migrants may carry pathogens or parasites encountered previously (e.g., Koprivnikar & Leung, 2015; Waldenström, Bensch, Kiboi, Hasselquist, & Ottosson, 2002) or be particularly vulnerable to infections during migration (e.g., Figuerola & Green, 2000). However, in a previous study on the same system, we measured haptoglobin concentration, an acute phase protein that indicates current infections, and found no evidence that migrants suffer more from current infections than residents, but rather the opposite (Eikenaar & Hegemann, 2016). If oxidative bursts would cause an association between AOX and BKA, also in the residents, these two parameters should have been negatively correlated, which they were not. Third, this alternative explanation would require strong variation in the need for oxidative burst among migrants. As all migrants were caught in the same area and in a previous study, no temporal pattern in migrating blackbirds' BKA was observed (Eikenaar & Hegemann, 2016), such variation cannot have been very large.

To conclude, our results suggest the existence of a physiological trade‐off between investment in baseline innate immune function and nonenzymatic antioxidant defenses that only becomes evident during the physiologically demanding migration season. Although we cannot provide experimental proof and hence no unequivocal evidence for this trade‐off, to the best of our knowledge, no such conclusive evidence exists for any taxonomic group nor does it exist for any other physiological system. Our study thereby is the first to provide an indication for the existence of a trade‐off between immune function and antioxidant defense. For migrating birds, such a trade‐off would very likely carry costs in terms of impaired health or increased oxidative damage. These costs could also represent one of the proximate causes of partial migration, that is the trade‐off between antioxidant defense and immune defense may affect the life‐history decision whether or not to migrate.

## CONFLICT OF INTEREST

None declared.

## AUTHOR CONTRIBUTIONS

CE conceived of the study and collected the data. AH and CI did all laboratory work. CE, CI, and AH together wrote the manuscript.

## DATA ACCESSIBILITY

The data belonging to this paper will be uploaded to Dryad.

## References

[ece33756-bib-0001] Alonso‐Alvarez, C. , & Ferrer, M. (2001). A biochemical study of fasting, subfeeding, and recovery processes in yellow‐legged gulls. Physiological and Biochemical Zoology, 74, 703–713. https://doi.org/10.1086/322932 11517455 10.1086/322932

[ece33756-bib-0002] Alonso‐Álvarez, C. , Pérez‐Rodriguez, L. , García, J. T. , Viñuela, J. , & Mateo, R. (2010). Age and breeding effort as sources of individual variability in oxidative stress markers in a bird species. Physiological and Biochemical Zoology, 83, 110–118. https://doi.org/10.1086/605395 19922287 10.1086/605395

[ece33756-bib-0003] Bairlein, F. , Fritz, J. , Scope, A. , Schwendenwein, I. , Stanclova, G. , van Dijk, G. , … Dittami, J. (2015). Energy expenditure and metabolic changes of free‐flying migrating northern bald ibis. PLoS ONE, 10(9), e0134433 https://doi.org/10.1371/journal.pone.0134433 26376193 10.1371/journal.pone.0134433 PMC4573986

[ece33756-bib-0004] Benzie, I. F. F. , & Strain, J. J. (1996). The ferric reducing ability of plasma (FRAP) as a measure of antioxidant power: The FRAP assay. Analytical Biochemistry, 239, 70–76. https://doi.org/10.1006/abio.1996.0292 8660627 10.1006/abio.1996.0292

[ece33756-bib-0005] Bolser, J. A. , Alan, R. R. , Smith, A. D. , Li, L. , Seeram, N. P. , & McWilliams, S. R. (2013). Birds select fruits with more anthocyanins and phenolic compounds during autumn migration. Wilson Journal of Ornithology, 125, 97–108. https://doi.org/10.1676/12-057.1

[ece33756-bib-0006] Buehler, D. M. , & Piersma, T. (2008). Travelling on a budget: Predictions and ecological evidence for bottlenecks in the annual cycle of long‐distance migrants. Philosophical Transactions of the Royal Society of London. Series B, Biological Sciences, 363, 247–266. https://doi.org/10.1098/rstb.2007.2138 17638692 10.1098/rstb.2007.2138 PMC2606749

[ece33756-bib-0007] Buehler, D. M. , Tieleman, B. I. , & Piersma, T. (2010). How do migratory species stay healthy over the annual cycle? A conceptual model for immune function and for resistance to disease. Integrative and Comparative Biology, 50, 346–357. https://doi.org/10.1093/icb/icq055 21558209 10.1093/icb/icq055

[ece33756-bib-0008] Chen, P. , Ma, Q. G. , Ji, C. , Zhang, J. Y. , Zhao, L. H. , Zhang, Y. , & Jie, Y. Z. (2011). Dietary lipoic acid influences antioxidant capability and oxidative status of broilers. International Journal of Molecular Sciences, 12, 8476–8488. https://doi.org/10.3390/ijms12128476 22272085 10.3390/ijms12128476 PMC3257082

[ece33756-bib-0009] Cohen, A. , Klasing, K. , & Ricklefs, R. (2007). Measuring circulating antioxidants in wild birds. Comparative Biochemistry and Physiology – Part B, 147, 110–121. https://doi.org/10.1016/j.cbpb.2006.12.015 10.1016/j.cbpb.2006.12.015 17303461

[ece33756-bib-0010] Costantini, D. (2014). Oxidative stress and hormesis in evolutionary ecology and physiology: A marriage between mechanistic and evolutionary approaches. Berlin, Germany: Springer https://doi.org/10.1007/978-3-642-54663-1

[ece33756-bib-0011] Costantini, D. , Cardinale, M. , & Carere, C. (2007). Oxidative damage and anti‐oxidant capacity in two migratory bird species at a stop‐over site. Comparative Biochemistry and Physiology Part C: Pharmacology, Toxicology and Endocrinology, 144, 363–371. 10.1016/j.cbpc.2006.11.005 17218158

[ece33756-bib-0012] Cram, D. L. , Blount, J. D. , York, J. E. , & Young, A. E. (2015). Immune response in a wild bird is predicted by oxidative status, but does not cause oxidative stress. PLoS ONE, 10(3), e0122421 https://doi.org/10.1371/journal.pone.0122421 25815888 10.1371/journal.pone.0122421 PMC4376632

[ece33756-bib-0013] Dierschke, J. , Dierschke, V. , Hüppop, K. , Hüppop, O. , & Jachmann, K. F. (2011). Die Vogelwelt der Insel Helgoland. Helgoland, Germany: OAG Helgoland.

[ece33756-bib-0014] Eikenaar, C. , & Hegemann, A. (2016). Migratory common blackbirds have lower innate immune function during autumn migration than resident conspecifics. Biology Letters, 12, 20160078 https://doi.org/10.1098/rsbl.2016.0078 27029839 10.1098/rsbl.2016.0078 PMC4843231

[ece33756-bib-0015] Eikenaar, C. , Källstig, E. , Andersson, M. N. , Herrera‐Dueñas, A. , & Isaksson, C. (2017). Oxidative challenges of avian migration: A comparative field study on a partial migrant. Physiological and Biochemical Zoology, 90, 223–229. https://doi.org/10.1086/689191 28277962 10.1086/689191

[ece33756-bib-0016] Eikenaar, C. , Müller, F. , Klinner, T. , & Bairlein, F. (2015). Baseline corticosterone levels are higher in migrating than sedentary common blackbirds in autumn, but not in spring. General and Comparative Endocrinology, 224, 121–125. https://doi.org/10.1016/j.ygcen.2015.07.003 26163918 10.1016/j.ygcen.2015.07.003

[ece33756-bib-0017] Erickson, K. L. , Median, E. A. , & Hubbard, N. E. (2000). Micronutrients and innate immunity. Journal of Infectious Diseases, 182, S5–S10. https://doi.org/10.1086/315922 10944478 10.1086/315922

[ece33756-bib-0018] Evans, E. , & Halliwell, B. (2001). Micronutrients: Oxidant/antioxdiant status. British Journal of Nutrition, 85, S67–S74. https://doi.org/10.1079/BJN2000296 11509092

[ece33756-bib-0019] Figuerola, J. , & Green, A. J. (2000). Haematozoan parasites and migratory behaviour in waterfowl. Evolutionary Ecology, 14, 143–153. https://doi.org/10.1023/A:1011009419264

[ece33756-bib-0020] French, S. S. , & Neuman‐Lee, L. A. (2012). Improved ex vivo method for microbiocidal activity across vertebrate species. Biology Open, 1, 482–487. https://doi.org/10.1242/bio.2012919 23213440 10.1242/bio.2012919 PMC3507210

[ece33756-bib-0021] Gardner, W. H. (1979). Lipid hydroperoxide reactivity with proteins and amino acids: A review. Journal of Agriculture and Food Chemistry, 27, 220–229. https://doi.org/10.1021/jf60222a034

[ece33756-bib-0022] Geiger, S. , Kauffmann, M. , Le Maho, Y. , Robin, J. P. , & Criscuolo, F. (2012). Of the importance of metabolic phases in the understanding of oxidative stress in prolonged fasting and refeeding. Physiological and Biochemical Zoology, 85, 415–420. 22705491 10.1086/666364

[ece33756-bib-0023] Gray, J. I. (1978). Measurement of lipid oxidation: A review. Journal of the American Oil Chemists Society, 55, 539–546. https://doi.org/10.1007/BF02668066

[ece33756-bib-0024] Griboff, J. , Morales, D. , Bertrand, L. , Bonansea, R. I. , Monferrán, M. V. , Asis, R. , … Amé, M. V. (2014). Oxidative stress response induced by atrazine in Palaemonetes argentinus: The protective effect of vitamin E. Ecotoxicology and Environmental Safety, 108, 1–8. https://doi.org/10.1016/j.ecoenv.2014.06.025 25038265 10.1016/j.ecoenv.2014.06.025

[ece33756-bib-0025] Hegemann, A. , Marra, P. P. , & Tieleman, B. I. (2015). Causes and consequences of partial migration in a passerine bird. The American Naturalist, 186, 531–546. https://doi.org/10.1086/682667 10.1086/682667 26655576

[ece33756-bib-0026] Hegemann, A. , Matson, K. D. , Both, C. , & Tieleman, B. I. (2012). Immune function in a free‐living bird varies over the annual cycle, but seasonal patterns differ between years. Oecologia, 170, 605–618. https://doi.org/10.1007/s00442-012-2339-3 22562421 10.1007/s00442-012-2339-3 PMC3470818

[ece33756-bib-0027] Hegemann, A. , Matson, K. D. , Flinks, H. , & Tieleman, B. I. (2013). Offspring pay sooner, parents pay later: Experimental manipulation of body mass reveals trade‐offs between immune function, reproduction and survival. Frontiers in Zoology, 10, 77. 24344978 10.1186/1742-9994-10-77 PMC3878409

[ece33756-bib-0028] Isaksson, C. , Sheldon, B. C. , & Uller, T. (2011). The challenges of integrating oxidative stress into life‐history biology. BioScience, 61, 194–202. https://doi.org/10.1525/bio.2011.61.3.5

[ece33756-bib-0029] Jenni‐Eiermann, S. , Jenni, L. , Smith, S. , & Costantini, D. (2014). Oxidative stress in endurance flight: And unconsidered factor in bird migration. PLoS ONE, 9(5), e97650 https://doi.org/10.1371/journal.pone.0097650 24830743 10.1371/journal.pone.0097650 PMC4022615

[ece33756-bib-0030] Kaiser, A. (1993). A new multi‐category classification of subcutaneous fat deposits of songbirds. Journal of Field Ornithology, 64, 246–255.

[ece33756-bib-0031] Kilgas, P. , Tilgar, V. , Külavee, R. , Saks, L. , Hõrak, P. , & Mänd, R. (2010). Antioxidant protection, immune function and growth of nestling great tits *Parus major* in relation to within‐brood hierarchy. Comparative Biochemistry and Physiology – Part B, 157, 288–293. https://doi.org/10.1016/j.cbpb.2010.07.002 10.1016/j.cbpb.2010.07.002 20647049

[ece33756-bib-0032] Kolmstetter, C. M. , & Ramsay, E. C. (2000). Effects of feeding on plasma uric acid and urea concentrations in blackfooted Penguins (*Spheniscus demersus*). Journal of Avian Medicine and Surgery, 14, 177–179. https://doi.org/10.1647/1082-6742(2000)014[0177:EOFOPU]2.0.CO;2

[ece33756-bib-0033] Koprivnikar, J. , & Leung, T. L. F. (2015). Flying with diverse passengers: Greater richness of parasitic nematodes in migratory birds. Oikos, 124, 399–405. https://doi.org/10.1111/oik.01799

[ece33756-bib-0134] Matson, K. D. , Ricklefs, R. E. , & Klasing, K. C. (2005). A hemolysis‐hemagglutination assay for characterizing constitutive innate humoral immunity in wild and domestic birds. Dev. Comp. Immunol, 29, 275–286. 15572075 10.1016/j.dci.2004.07.006

[ece33756-bib-0034] Matson, K. , Tieleman, B. I. , & Klasing, K. C. (2006). Capture stress and the bactericidal competence of blood and plasma in five species of tropical birds. Physiological and Biochemical Zoology, 79, 556–564. https://doi.org/10.1086/501057 16691521 10.1086/501057

[ece33756-bib-0035] Millet, S. , Bennett, J. , Lee, K. A. , Hau, M. , & Klasing, K. C. (2007). Quantifying and comparing constitutive immunity across avian species. Developmental and Comparative Immunology, 31, 188–201. https://doi.org/10.1016/j.dci.2006.05.013 16870251 10.1016/j.dci.2006.05.013

[ece33756-bib-0036] Møller, A. P. , & Erritzoe, J. (1998). Host immune defence and migration in birds. Evolutionary Ecology, 12, 945–953. https://doi.org/10.1023/A:1006516222343

[ece33756-bib-0037] Monaghan, P. , Metcalfe, N. B. , & Torres, R. (2009). Oxidative stress as a mediator of life‐history trade‐offs: Mechanisms, measurements, and interpretation. Ecology Letters, 12, 75–92. https://doi.org/10.1111/j.1461-0248.2008.01258.x 19016828 10.1111/j.1461-0248.2008.01258.x

[ece33756-bib-0038] Nebel, S. , Bauchinger, U. , Buehler, D. M. , Langlois, L. A. , Boyles, M. , Gerson, A. R. , … Guglielmo, C. G. (2012). Constitutive immune function in European starlings, *Sturnus vulgaris*, is decreased immediately after an endurance flight in a wind tunnel. Journal of Experimental Biology, 215, 272–278. https://doi.org/10.1242/jeb.057885 22189771 10.1242/jeb.057885

[ece33756-bib-0039] Ochsenbein, A. F. , & Zinkernagel, R. M. (2000). Natural antibodies and complement link innate and acquired immunity. Immunology Today, 21, 624–630. https://doi.org/10.1016/S0167-5699(00)01754-0 11114423 10.1016/s0167-5699(00)01754-0

[ece33756-bib-0040] Ouyang, J. Q. , Lendvai, Á. Z. , Moore, I. T. , Bonier, F. , & Haussmann, M. F. (2016). Do hormones, telomere lengths, and oxidative stress form an integrated phenotype? A case study in free‐living tree swallows. Integrative and Comparative Biology, 56, 138–145. https://doi.org/10.1093/icb/icw044 27252220 10.1093/icb/icw044 PMC6592418

[ece33756-bib-0041] Pérez‐Rodríguez, L. , Romero‐Haro, A. A. , Sternalski, A. , Muriel, J. , Mougeot, F. , Gil, D. , & Alonso‐Alvarez, C. (2015). Measuring oxidative stress: The confounding effect of lipid concentration in measures of lipid peroxidation. Physiological and Biochemical Zoology, 88, 345–351. https://doi.org/10.1086/680688 25860832 10.1086/680688

[ece33756-bib-0042] Roff, D. A. (1992). The evolution of life histories. New York, NY: Chapman and Hall.

[ece33756-bib-0043] Romero‐Haro, A. A. , & Alonso‐Alvarez, C. (2014). Covariation in oxidative stress markers in the blood of nestling and adult birds. Physiological and Biochemical Zoology, 87, 353–362. https://doi.org/10.1086/674432 24642552 10.1086/674432

[ece33756-bib-0044] Sacher, T. (2009). Genetic differentiation and migration behaviour of an island population of the common blackbird (Turdus merula). PhD thesis, Wilhemshaven.

[ece33756-bib-0045] Schielzeth, H. (2010). Simple means to improve the interpretability of regression coefficients. Methods in Ecology and Evolution, 1, 103–113. https://doi.org/10.1111/j.2041-210X.2010.00012.x

[ece33756-bib-0046] Schmaljohann, H. , Fox, J. W. , & Bairlein, F. (2012). Phenotypic response to environmental cues, orientation and migration costs in songbirds flying halfway around the world. Animal Behavior, 84, 623–640. https://doi.org/10.1016/j.anbehav.2012.06.018

[ece33756-bib-0047] Schwenke, R. A. , Lazzaro, B. P. , & Wolfner, M. F. (2016). Reproduction‐immunity trade‐offs in insects. Annual Review of Entomology, 61, 239–256. https://doi.org/10.1146/annurev-ento-010715-023924 10.1146/annurev-ento-010715-023924 PMC5231921 26667271

[ece33756-bib-0048] Skrip, M. , Bauchinger, U. , Goymann, W. , Fusani, L. , Cardinale, M. , Alan, R. R. , & McWilliams, S. R. (2015). Migrating songbirds at stopover prepare for, and recover from, oxidative challenges posed by long‐distance flight. Ecology and Evolution, 5, 3198–3209. https://doi.org/10.1002/ece3.1601 26355277 10.1002/ece3.1601 PMC4559061

[ece33756-bib-0049] Skrip, M. M. , Seeram, N. P. , Yuan, T. , Ma, H. , & McWilliams, S. R. (2016). Dietary antioxidants and flight exercise in female birds affect allocation of nutrients to eggs: How carry‐over effects work. Journal of Experimental Biology, 219, 2716–2725. https://doi.org/10.1242/jeb.137802 27582563 10.1242/jeb.137802

[ece33756-bib-0050] Stearns, S. C. (1992). The evolution of life histories. Oxford: Oxford University Press.

[ece33756-bib-0051] Stinefelt, B. , Leonard, S. S. , Blemings, K. P. , Shi, X. , & Klandorf, H. (2005). Free radical scavenging, DNA protection, and inhibition of lipid peroxidation mediated by uric acid. Annals of Clinical and Laboratory Science, 35, 37–45. 15830708

[ece33756-bib-0052] Svensson, L. (1992). Identification guide to European passerines, 4th ed. Stockholm, Sweden: L. Svensson.

[ece33756-bib-0053] Tieleman, B. I. , Williams, J. B. , Ricklefs, R. E. , & Klasing, K. C. (2005). Constitutive innate immunity is a component of the paceof‐ life syndrome in tropical birds. Proceedings of the Royal Society of London, Series B: Biological Sciences, 272, 1715–1720. https://doi.org/10.1098/rspb.2005.3155 16087427 10.1098/rspb.2005.3155 PMC1559858

[ece33756-bib-0054] Van de Crommenacker, J. , Komdeur, J. , Burke, T. , & Richardson, D. S. (2011). Spatio‐temporal variation in territory quality and oxidative status: A natural experiment in the Seychelles warbler (*Acrocephalus sechellensis*). Journal of Animal Ecology, 80, 668–680. https://doi.org/10.1111/j.1365-2656.2010.01792.x 21198588 10.1111/j.1365-2656.2010.01792.x PMC3107423

[ece33756-bib-0055] van Noordwijk, A. J. , & de Jong, G. (1986). Acquisition and allocation of resources: Their influence on variation in life history tactics. The American Naturalist, 128, 137–142. https://doi.org/10.1086/284547

[ece33756-bib-0056] Von Schantz, T. , Bensch, S. , Grahn, M. , Hasselquist, D. , & Wittzell, H. (1999). Good genes, oxidative stress and condition‐dependent sexual signals. Proceedings of the Royal Society of London, Series B: Biological Sciences, 266, 1–12. https://doi.org/10.1098/rspb.1999.0597 10081154 10.1098/rspb.1999.0597 PMC1689644

[ece33756-bib-0057] Waldenström, J. , Bensch, S. , Kiboi, S. , Hasselquist, D. , & Ottosson, U. (2002). Cross‐species infection of blood parasites between resident and migratory songbirds in Africa. Molecular Ecology, 11, 1545–1554. https://doi.org/10.1046/j.1365-294X.2002.01523.x 12144673 10.1046/j.1365-294x.2002.01523.x

[ece33756-bib-0058] Wikelski, M. , Tarlow, E. M. , Raim, A. , Diehl, R. H. , Larkin, R. P. , & Visser, G. H. (2003). Costs of migration in free‐flying songbirds. Nature, 423, 704–770. https://doi.org/10.1038/423704a 12802324 10.1038/423704a

[ece33756-bib-0059] Wone, B. , Ojha, J. , Contreras, H. , & Davidowitz, D. (2014). More is not always better: A hidden cost of the flight‐fecundity trade‐off in the hawk moth, *Manduca sexta* . FASEB Journal, 28(S1100), 2.24385569

[ece33756-bib-0060] Zera, A. J. , & Harshman, L. G. (2001). The physiology of life‐history trade‐offs in animals. Annual Review of Ecology and Systematics, 32, 95–126. https://doi.org/10.1146/annurev.ecolsys.32.081501.114006

