# Supplementary material for: A hidden cost of migration? Innate immune function versus antioxidant defense
Source: Ecol Evol. 2018 Feb 7;8(5):2721–8. doi: 10.1002/ece3.3756 (PMC5838071; doi:10.1002/ece3.3756)
Supplement: Supplementary file 1 [file ECE3-8-2721-s001.docx]

**Supporting information**

Figure S1. The linear regression of uric acid (UA) concentration against log_10_-transformed total non-enzymatic antioxidant capacity (AOX) in common blackbirds. Beta and SE: 0.014 ± 0.001, t = 13.5, p < 0.001, n = 55. R-squared = 0.78.

Plasmatic lipid concentrations can have confounding effects on measures of lipid peroxidation (Pérez-Rodriguez et al. 2015). We therefore calculated a fatty acid (FA) peroxidation index (after Eikenaar et al. (2017), see also Appendix for details), which takes into account the FA concentration and how peroxidizable the unsaturated FAs are, i.e. the more double bonds the easier the FA are peroxidized. In our sample, however, MDA concentration was not related to FA peroxidation index (see Fig. S2). Hence, there was no need to correct MDA concentration for FA peroxidation index.

Figure S2. The linear regression of fatty acid (FA) peroxidation index against log_10_-transformed malondialdehyde (MDA) concentration in common blackbirds. Beta and SE: -0.008 ± 0.035, T = -0.24, p = 0.81, n = 55. R-squared = 0.001. To reduce the number of zeros behind the decimal point in the parameter estimate, FA peroxidation index was divided by 1000. Note that the figure shows original values of this index.

**References**

Pérez-Rodriguez, L., Romero-Haro, A.A., Sternalski, A., Muriel, J., Mougeot, F., Gil, D., et al. (2015). Measuring oxidative stress: the confounding effect of lipid concentration in measures of lipid peroxidation. *Phys. Biochem. Zool.*, **88**, 345-351.

Eikenaar, C., Källstig, E. Andersson, M. N., Herrera-Dueñas, A. and Isaksson, C. (2017). Oxidative challenges of avian migration: a comparative field study on a partial migrant. *Phys. Biochem. Zool.*, **90**, 223-229.
