# Supplementary material for: A hidden cost of migration? Innate immune function versus antioxidant defense
Source: Ecol Evol. 2018 Feb 7;8(5):2721–8. doi: 10.1002/ece3.3756 (PMC5838071; doi:10.1002/ece3.3756)
Supplement: Supplementary file 2 [file ECE3-8-2721-s002.docx]

**Appendix S1**

All samples were randomized before lab work, which was conducted blind with respect to whether individuals were migrants or residents. All chemicals used were purchased from Sigma-Aldrich (Stockholm, Sweden), unless stated otherwise.

Bacteria killing capacity (BKA)

We quantified the microbial killing capacity (against *E. coli*) of plasma following the method described by French and Neumann-Lee (2012) with a few modifications. We used 3 µl of frozen plasma and mixed it in 4 µl of 10^5 *E. coli* solution. Plates were incubated at 37°C for 12 hours, subsequently vortexed for 1 min at 100 rpm, and read at 600 nm using a microplate reader. To calculate bacterial killing ability we first subtracted background absorbance readings from the absorbance readings (i.e., 12 hour readings). We calculated the % of *E. coli* killed relative to the growth of *E. coli* in the positive controls (wells containing only bacterial and broth, run in quadruple per plate) as one minus the mean absorbance for each sample, divided by the mean absorbance for the positive controls and multiplied by 100. We used 4 negative controls per plate to ensure that there was no contamination.

Total non-enzymatic antioxidant capacity (AOX)

AOX was measured using the ferric reducing antioxidant power (FRAP) assay, which gives the overall reducing potential i.e. non-enzymatic antioxidant potential of the sample (Benzie and Strain 1996). Briefly, 5 µl of plasma was diluted 1:8 with ddH_2_O, 20 µl of the diluted plasma sample was then incubated with 150 µl working solution prepared freshly each six hours (Sodium acetate trihydrate + 2, 4, 6-Tris (2-pyridyl)-s-triazine (TPTZ) + Iron (III) chloride hexahydrate (FeCl_3_ - 6H_2_O); 10:1:1) for 20 min at room temperature. Immediately following incubation, the colour generated from the reduction of Fe^3+^ (ferric) to Fe^2+^ (ferrous) was measured on a FLUOstar OMEGA (BMG LABTECH) plate reader at 593 nm. The data obtained were compared with a standard curve made from standards of known Fe^2+^ concentration (Iron (II) sulphate heptahydrate (FeSO_4_ – 7H_2_O)) prepared freshly each day. AOX was measured in duplicates with inter- and intra-assay variations of 4.44 and 3.84, respectively.

Uric acid (UA)

UA concentrations were assessed in 5 μl of plasma using a commercial kit from SPINREACT (Sant Esteve de Bas, Spain). Following the manufacturer’s instructions, the red colour, formed after enzymatic (uricase and peroxidase) reactions with uric acid, was measured by a FLUOstar OMEGA (BMG LABTECH) plate reader at 520 nm. Assays were run in duplicates with inter- and intra-assay variations of 3.47and 4.25, respectively.

Malondialdehyde (MDA)

MDA is a secondary product of peroxidation of polyunsaturated fatty acids (Gardner 1979), and is a commonly used biomarker of lipid peroxidation. MDA concentration was measured according to Eikenaar et al. (2016) by coupled gas chromatography and electron ionization mass spectrometry (GC/EI/MS) analysis after derivatization with O-(2,3,4,5,6-pentafluorbenzyl) hydroxylamine hydrochloride (PFBHA⋅HCl). 15 µl of plasma was mixed with 50 µl PFB solution (1 mM in sodium acetate buffer, pH 5.0) and the micro reaction was conducted for 1h at room tempreature. The resulted MDA-bis-(PFB-oxime) derivatives were extracted by 300 µl n-hexane containing 1.65 pg/µl of 3-bromofluorobenzene as internal standard, and analyzed by an Agilent 5975 mass-selective detector coupled to an Agilent 6890 gas chromatograph. A non-polar capillary column (HP-5MS: 30 m x 0.25 mm i.d., and 0.25 µm film thickness; J&W Scientific, USA) was used for GC/MS analysis, and two characteristic ions at *m*/*z* 181 and 250 were measured under selected ion monitoring mode to quantify the target derivatives. The GC oven was programmed from 60 °C for 1 min, at a rate of 15 °C/min to 150 °C, and then at a rate of 10 °C/min to 270 °C, held for 5 min.

Fatty acids (FAs)

FAs were extracted and subsequently analyzed using GC/MS according to previously established methods (Andersson et al. 2015). A total lipid extraction of 5 µl plasma was conducted for 1 h at RT using 50 µl chloroform:methanol (2:1 v/v) containing 1.67 µg of the internal standard methyl *cis*-10-heptadecenoate (purity >99%, Aldrich). Samples were then dried under N_2_ after which base methanolysis, using 100 µl 0.5 M KOH/Me, was conducted for 1h at 40 °C to convert fatty-acyl moieties into corresponding FA methyl esters (FAMEs). The reaction was terminated by adding 100 µl 0.5 M HCl/Me, and 300 µl *n*-heptane (purity >99%, VWR Prolabo) was added to extract the resulting FAMEs. Heptane extracts were washed twice with 200 µl de-ionized H_2_O, and residual water removed using anhydrous sodium sulfate. Samples were analyzed using an Agilent 5975 MS coupled to an Agilent 6890 GC equipped with an HP-INNOWax PEG column (30 m, 0.25 mm id, 0.25 μm film thickness; Agilent). Helium was used as carrier gas at a flow of 1 ml/min. The oven temperature was programmed to 80 °C for 1 min, then increased by 10 °C /min to 230 °C and held for 20 min. Twenty FAMEs were identified by comparing mass spectra and retention times with those of synthetic standards (Supelco 37-Component FAME mix) and their concentrations quantified based on the abundance of the internal standard. Since MDA concentrations correlate with absolute concentrations of peroxidizable FAs (Pérez-Rodriguez et al. 2015) rather than their relative abundance, we modified the percentage-based equation described by e.g. Pamplona et al. (1998), and calculated the peroxidation index as: peroxidation index = [(conc. monoenoic acid (in µg FA/µl plasma) × 0.025) + (conc. dienoic × 1) + (conc. trienoic × 2) + (conc. tetraenoic × 4) + (conc. pentaenoic × 6) + (conc. hexaenoic × 8)].

**References**

French, S.S. and Neumann-Lee, L.A. (2012). Improved ex vivo method for microbiocidal activity across vertebrate species. *Biol. Open*, **1** 482-487.

Benzie, I.F.F. and Strain, J.J. (1996). The ferric reducing ability of plasma (FRAP) as a measure of antioxidant power: the FRAP assay. *Analyt. Biochem*. **239**, 70-76.

Gardner, W.H. (1979). Lipid hydroperoxide reactivity with proteins and amino acids: a review. *J. Agric. Food Chem.* **27**, 220-229.

Eikenaar, C., Jönsson, J., Fritzsch, A., Wang, H.L. and Isaksson, C. (2016). Migratory refueling affects non-enzymatic antioxidant capacity, but does not increase lipid peroxidation. *Physiol. Behav.* **158**, 26-32.

Andersson, M.N., Wang, H.L., Nord, A., Salmón,P. and Isaksson, C. (2015). Composition of physiologically important fatty acids in great tits differs between urban and rural populations on a seasonal basis. *Front. Ecol. Evol.* **3**:93.

Pérez-Rodriguez, L., Romero-Haro, A.A., Sternalski, A., Muriel, J., Mougeot, F., Gil, D., et al. (2015). Measuring oxidative stress: the confounding effect of lipid concentration in measures of lipid peroxidation. *Phys. Biochem. Zool.* **88**, 345-351.

Pamplona, R., Portero-Otín, M., Riba, D., Ruiz, C., Prat, J., Bellmunt, M.J., et al. (1998). Mitochondrial membrane peroxidizability index is inversely related to maximum life-span in mammals. *J. Lipid Res.* **39**, 1989-1994.
